# Exemption of Certain Parts of the Delta of the Mississippi, from Autumnal Diseases

**Published:** 1835

**Authors:** 


### X.—EXEMPTION OF CERTAIN PARTS OF THE DELTA OF THE MISSISSIPPI, FROM AUTUMNAL DISEASES.

In an interview which we lately had with the very intelligent and respectable Dr. Cartwright, of Natchez, he pointed out certain parts of the Delta of the Mississippi, which are proverbially exempt from summer and autumnal diseases, and deserve, in his opinion, to be classed among the very healthiest portions of the Union. They are the banks of the numerous bayous and lagunes (obsolete mouths of the Mississippi,) between the thirty-first degree of latitude and the Gulf of Mexico, lying west of Lafourche. The water in these bayous, five or six in number, is stagnant. On either margin there is a slip of land from a quarter to half a mile in width, that is sufficiently high to be cultivated, back of this slip is a cypress swamp, then a shaking prairie, and then a lake, the banks of which are uninhabitable. The water of the bayous although stagnant is clear, and almost hidden by aquatic plants, one species of which, the most abundant, is unconnected with the soil and floats in the water—having a knotted horizontal root, resembling that of the cane. Another appears to be the *Pistia Stratiotes* of the botanists.

From observations and inquiries made on the spot, and on similar localities in other parts of Louisiana, where the currents of the bayous prevent the growth of these vegetables, Dr. C. has come to the conclusion, that the sole cause of the great salubrity of the places referred to, is their action on the waters and the air; and, in accordance with these views, has taken measures to introduce them into lakes further up the Mississippi, the shores of which are now infested with bilious fevers.

